# Dataset and response surface modeling tools for data-driven optimization of a functional cupuassu (*Theobroma grandiflorum*) cremogenate

**DOI:** 10.1016/j.dib.2026.113305

**Published:** 2026-10-01

**Authors:** Paola A. García-Rincón, Andrés F. Bahamón-Monje, Gentil․ A. Collazos-Escobar, Nelson Gutiérrez-Guzmán

**Affiliations:** a Grupo de investigación de Biotecnología y Control de Calidad de Alimentos, Universidad de la Amazonia, Florencia-Caquetá, 180001, Colombia; b Centro Surcolombiano de Investigación en Café (CESURCAFÉ), Departamento de Ingeniería Agrícola, Universidad Surcolombiana, Neiva-Huila, 410001, Colombia; c University Institute of Food Engineering-FoodUPV, Universitat Politècnica de València, Camino de Vera s/n, València, 46022, Spain; d Departamento de Ingeniería Agroindustrial, Universidad Surcolombiana, Neiva, Huila, 410001, Colombia

**Keywords:** Cupuassu, Functional fruit cremogenate, *Lactobacillus casei* ATCC 393, Response surface methodology, Model-based optimization

## Abstract

This article reports an experimental dataset for the optimization of a functional cupuassu (*Theobroma grandiflorum*) cremogenate formulated with *Lactobacillus casei* ATCC 393 and inulin. The data were obtained using a central composite design and computational Response Surface Methodology (RSM) tools to assess the multivariate effects of probiotic biomass (2.0, 2.25, and 2.5%), inulin concentration (2, 3, and 4%), and homogenization time (5, 7.5, and 10 min) on key quality attributes of the cupuassu-based cremogenate. The measured response variables included moisture content (%wet basis), water activity, viscosity (cP), hydrogen potential (pH), soluble solids content (°Brix), particle-size distribution (D10, D50, and D90; µm), zeta potential (mV), and probiotic viability determined by colony counting (CC, log CFU/g). The dataset provides a robust experimental framework for elucidating the interactions between formulation and processing variables affecting the quality attributes of a functional cupuassu-based cremogenate. Furthermore, this work presents a computational workflow implemented in MATLAB for response surface modeling, statistical prediction, and process optimization. The scripts support the fitting of second-order polynomial models, visualization of response surfaces, analysis of factor interactions, surface response optimization, and prediction of optimal formulation conditions. These computational tools enhance reproducibility and offer a flexible framework for extending optimization strategies to other functional food systems. The integration of the experimental and computational datasets constitutes a reliable basis for the rational design of probiotic fruit-based products and for the application of advanced statistical optimization approaches in food process engineering.

## Graphical abstract

**Figure:**
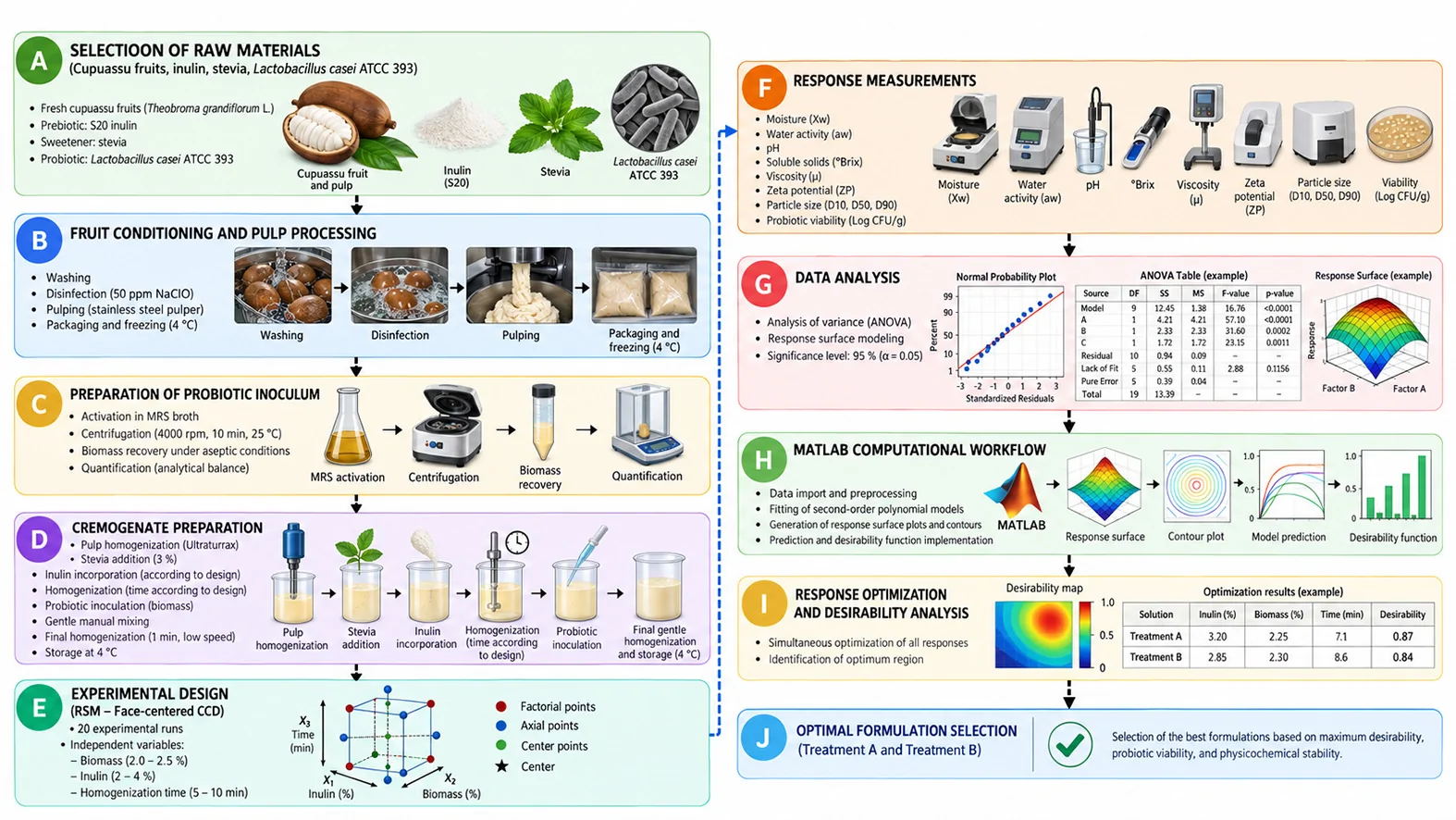

Specifications Table

**Table:** 

Subject	Food Engineering
Specific subject area	Food technology, Food Science
Type of data	Excel file (Dataset for cupuassu-based cremogenate, including experimental process conditions, replicate runs, and measured response variables related to quality parameters). StatGraphics files (Analysis of variance-ANOVA and statistical modeling outputs). MATLAB file (Computational modeling scripts for multiple linear regression, response surface analysis, and optimization). Figures (Response surface plots and schematic flowchart illustrating the experimental design and dataset generation workflow).
Data collection	Moisture content (AOAC 930.15/1990), water activity (AOAC 978.18), °brix (AOAC 932.12) and pH (AOAC 942.15/970.21). Viscosity, particle-size distribution (D10, D50 and D90), zeta potential and CC were determined following methods reported in scientific literature.
Data source location	The experimental dataset reported in this study was generated at the Centro Surcolombiano de Investigación en Café (CESURCAFÉ) of the Universidad Surcolombiana (Neiva, Huila-Colombia), in collaboration with the Biotechnology and Food Quality Control Laboratory of the Universidad de la Amazonia (Florencia, Caquetá-Colombia).
Data accessibility	Repository name: Mendeley Data Data identification number: 10.17632/rszrs6bg4f.4 Direct URL to data: https://data.mendeley.com/datasets/rszrs6bg4f/4
Related research article	None.

## 1 Value of the Data

• These data provide a comprehensive experimental framework for the formulation and process optimization of a cupuassu (*Theobroma grandiflorum*) fruit cremogenate enriched with *Lactobacillus casei* ATCC 393 and inulin as a prebiotic ingredient.
• The dataset supports the development of non-dairy functional fruit-based products by demonstrating that cupuassu pulp is a suitable matrix for maintaining probiotic viability above the minimum functional threshold of 10^6^ CFU/g.
• The experimental design enables the simultaneous assessment of formulation variables, including probiotic biomass, inulin concentration, and homogenization time, as well as their effects on physicochemical, rheological, colloidal, particle-size, and microbiological responses.
• These data are valuable for researchers and food technologists working in response surface methodology, process optimization, and the design of functional fruit-based products with improved technological and functional properties.
• The dataset and computational tools enable the identification of optimal processing conditions that balance probiotic survival, soluble solids, viscosity, pH, water activity, zeta potential, and particle-size distribution, thereby providing a basis for scale-up and industrial formulation.
• The dataset contributes to the valorization of Amazonian fruit resources, particularly cupuassu, by supporting their use in sustainable agro-industrial innovation and in the diversification of functional food products.

## 2 Background

Functional fruit-based foods have attracted increasing scientific and industrial attention as they combine the intrinsic nutritional value of plant matrices with bioactive or physiologically relevant ingredients. Their conceptual basis is rooted in the long-standing human pursuit of longevity and well-being, which has progressively evolved into the modern concept of functional foods as products capable of promoting health beyond basic nutrition [1]. In parallel, the fruit- and vegetable-based food sector has expanded considerably in recent years, driven by consumer demand for more natural products, reduced use of synthetic preservatives, and healthier dietary choices associated with lower risk of chronic disease [2]. In Latin American food culture, where juices and nectars are traditionally dominant forms of fruit consumption, there is increasing interest in developing alternative fruit-based products that diversify consumption patterns and increase annual per capita fruit intake.

Among these alternatives, fruit cremogenates represent an attractive technological and nutritional option. Cremogenates are obtained from fruit pulps or blends through mechanical disintegration processes such as milling, sieving, or ultra-homogenization, using the fruit’s own aqueous phase and preserving much of its native chemical composition [3]. Compared with conventional clarified juices or nectars, cremogenate retains a larger proportion of the original fruit matrix, including soluble solids, dietary fiber, organic acids, pectic substances, and colloidal components, thereby offering superior nutritional and structural functionality. This greater matrix integrity provides not only technological advantages, such as improved viscosity and suspension stability, but also aligns with current market trends favoring minimally processed functional foods that naturally preserve nutritional quality over time.

In this context, the Amazon region represents an important reservoir of underutilized fruits with high potential for functional food innovation. Among them, species of the genus *Theobroma* are of particular relevance in the Colombian and Brazilian Amazon. Cupuassu (*Theobroma grandiflorum*), a native fruit of the Brazilian Amazon also distributed in the Colombian Amazon, has emerged as a promising agro-industrial raw material due to its distinctive aroma, exotic sensory profile, and valuable nutritional composition [4]. Its pulp is rich in carbohydrates, soluble dietary fiber, pectic polysaccharides, and starch, and contains several compounds with physiological activity that support its use in the formulation of value-added functional products [5]. These characteristics make cupuassu particularly suitable for the development of fruit-based functional matrices with desirable technological and nutritional properties.

The incorporation of probiotics and prebiotics into fruit matrices offers a compelling alternative to traditional dairy-based functional foods. Plant-based probiotic systems are increasingly recognized for their potential to deliver health-promoting microorganisms in non-dairy vehicles, responding to the growing demand for vegan, lactose-free, and low-allergen products. Probiotic and prebiotic ingredients in vegetable matrices have been associated with several health benefits, including prevention of gastrointestinal disorders, modulation of intestinal microbiota, improvement of irritable bowel syndrome symptoms, and strengthening of immune function [6]. These effects depend strongly on both the concentration and the specific strain employed [7]. Among probiotic candidates, *Lactobacillus casei* has received particular attention due to its technological robustness and reported functional potential. *Lactobacillus casei* is a microaerophilic lactic acid bacterium naturally associated with fruits, vegetables, dairy products, and intestinal and vaginal mucosa [8]. It has been reported to contribute to cholesterol reduction, support intestinal microbial homeostasis, improve digestive function through organic acid production, and assist in immune regulation. Its physiological resilience and compatibility with plant matrices make *Lactobacillus casei* ATCC 393 a promising candidate for incorporation into acidic fruit systems. Likewise, among the most widely studied prebiotics, inulin is particularly relevant because of its combined nutritional and technological benefits. Beyond its recognized prebiotic effect, inulin can contribute to texture, mouthfeel, and colloidal stabilization, making it suitable for fruit-based formulations, either alone or in combination with probiotic microorganisms. However, a recent study by Kalagatur et al. [9] reported that the combined supplementation of inulin and β-glucan improved probiotic viability in Alphonso mango juice during refrigerated storage. This finding highlights the potential advantages of mixed prebiotic systems in fruit-based probiotic delivery vehicles. Therefore, the evaluation of different prebiotics, both individually and in combination, represents a relevant research direction for advancing the development of cupuassu-based cremogenates.

For a product to be considered probiotic, however, it must comply with established regulatory criteria regarding microbial viability. One of the principal requirements is the maintenance of a minimum viable probiotic population of 1 × 10^6^ CFU/g or CFU/mL throughout the product’s shelf life [10]. This represents a major technological challenge in fruit-based systems, since the acidic pH, oxygen exposure, and presence of native antimicrobial compounds in fruit matrices may compromise probiotic survival. Therefore, formulation and processing conditions must be carefully optimized to ensure both microbial viability and desirable product quality.

The development of stable and acceptable fruit cremogenate enriched with probiotics requires a rational formulation strategy supported by process modeling and optimization. Experimental optimization is widely recognized as an effective approach for improving product quality, supporting diversification in the food chain, and increasing the commercial and export potential of agro-industrial products [11]. In food product design, robust statistical and experimental tools are frequently employed to evaluate and optimize formulations involving multiple ingredients and interacting variables [12]. This task is especially relevant in research and development settings, where interdisciplinary decisions must balance technological feasibility, product quality, and economic efficiency. In products such as juices, nectars, purees, jams, yogurts, and cremogenates, optimal ingredient combinations are required to achieve desirable sensory, rheological, physical, and chemical characteristics while maintaining economic viability, particularly when one or more ingredients are costly [13]. Among optimization strategies, RSM is one of the most widely used and effective tools in food systems engineering [14]. RSM enables the simultaneous evaluation of multiple factors and their interactions, facilitating the identification of optimal processing and formulation conditions with reduced experimental effort [15]. In the food industry, this methodology has been successfully applied to optimize nutritional, sensory, caloric, and technological attributes in functional product development [16]. Its application is particularly valuable in complex colloidal systems such as fruit cremogenates, where composition and processing variables can significantly affect physicochemical stability, rheological behavior, and probiotic viability.

Therefore, this dataset applies RSM to optimize a cupuassu-based cremogenate supplemented with *Lactobacillus casei* ATCC 393 and inulin. The experimental design evaluates the effects of probiotic biomass, inulin concentration, and homogenization time on the physicochemical, rheological, colloidal, particle-size, and microbiological properties of the formulation. By integrating technological quality attributes with probiotic viability, the dataset provides a scientific basis for the development of innovative functional products derived from Amazonian fruits.

## 3 Data Description

The experimental dataset was organized into Excel files, Statgraphics outputs, and MATLAB scripts according to experimental runs, replicate measurements, and response variables.

**dataCremoGen.xlsx:** This Excel file contains the complete structured experimental dataset used for the formulation, statistical modeling, and optimization of a probiotic cupuassu (*Theobroma grandiflorum*) cremogenate enriched with *Lactobacillus casei* ATCC 393 and inulin. This spreadsheet constitutes the primary experimental matrix of the study and was designed to support reproducible response surface modeling, multivariate statistical analysis, and computational optimization.

The dataset was obtained using a face-centered central composite design (FCCD), implemented in Design-Expert software (Design-Expert V11, Stat-Ease, Inc.). This design was selected to assess the combined effects of three independent processing variables on the quality of the probiotic cupuassu-based cremogenate while minimizing the number of experimental runs and preserving statistical robustness [17]. The FCCD included 15 treatment combinations (Table 1) in which the center condition (2.25% biomass, 3% inulin, and 7.5 min homogenization time) was repeated six times to estimate pure error and support the assessment of curvature in the fitted second-order polynomial models. Therefore, the design matrix contained 20 experimental runs (Table 1) in total, but only 15 unique treatment combinations. Furthermore, each experimental run (n=20) was conducted in three replicates (20 × 3) to ensure reproducibility and to enable robust statistical analysis, resulting in a total of 60 independent runs.

**Table 1:** Formulation design of cupuassu-based cremogenate with probiotic addition.

Experimental run	Treatment	Biomass (%)	Inulin (%)	Time (min)
1	T1	2.0	4.0	10.0
2	T2	2.0	3.0	7.5
3	T3	2.25	3.0	10.0
4	T4	2.0	2.0	10.0
5	T5	2.25	3.0	7.5
6	T5	2.25	3.0	7.5
7	T5	2.25	3.0	7.5
8	T5	2.25	3.0	7.5
9	T5	2.25	3.0	7.5
10	T5	2.25	3.0	7.5
11	T6	2.25	3.0	5.0
12	T7	2.5	3.0	7.5
13	T8	2.0	4.0	5.0
14	T9	2.25	2.0	7.5
15	T10	2.0	2.0	5.0
16	T11	2.5	4.0	10.0
17	T12	2.25	4.0	7.5
18	T13	2.5	2.0	5.0
19	T14	2.5	4.0	5.0
20	T15	2.5	2.0	10.0

The independent variables evaluated in the design were:

• Biomass (%): probiotic biomass concentration of *Lactobacillus casei* ATCC 393, evaluated at 2.0, 2.25, and 2.5%.
• Inulin (%): prebiotic inulin concentration, evaluated at 2, 3, and 4%.
• Time (min): homogenization time during formulation, evaluated at 5, 7.5, and 10 min.

For each experimental run and replicates, a set of key physicochemical, rheological, colloidal, and microbiological response variables was measured to characterize product performance. The dependent variables recorded in the spreadsheet are, moisture content (%wet basis, %w.b.), water activity, hydrogen potential (pH), soluble solids content (°Brix), viscosity (cP), article diameter below which 10% of the sample volume is distributed D10 (µm), median particle diameter D50 (µm), particle diameter below which 90% of the sample volume is distributed D90 (µm), zeta potential (mV), viable probiotic cell concentration expressed as logarithm of colony-forming units per gram CC (log CFU/g).

For each response variable, an independent ANOVA was performed and the corresponding statistical outputs were compiled in individual Statgraphics files: **ANOVA_Moisture, ANOVA_WaterActivity, ANOVA_Viscosity, ANOVA_pH, ANOVA_°Brix, ANOVA_D10, ANOVA_D50, ANOVA_D90, ANOVA_ZetaPotential**, and **ANOVA_ColonyCounting**. These files contain the ANOVA results used to evaluate the statistical significance of the process variables (Biomass, inulin and time) on response variables. The experimental data obtained from the design of experiments and ANOVA results are summarized in Table 2, Table 3, which report mean values ± standard deviation of responses, significance levels and p-values used to identify the main process variables affecting each quality attribute of the cupuassu-based cremogenate.

**Table 2:** Experimental design and analysis of variance (ANOVA) results for the analysis of cupuassu (*Theobroma grandiflorum*)-based cremogenate supplemented with *Lactobacillus casei* ATCC 393 and inulin. The table summarizes the experimental runs, formulation and processing factors, measured response variables, and the corresponding ANOVA outputs used to evaluate the significance of model terms and factor interactions.

Treatment	Biomass (%)	Inulin (%)	Time (min)	Moisture content (%w.b.)	Water activity	Viscosity (cP)	pH	°Brix
T1	2	4	10	87.88±0.50	0.988±0.005	1400.67±9.87	3.51±0.02	15.17±0.25
T2	2	3	7.5	85.84±0.90	0.988±0.007	1358.00±8.72	3.53±0.03	14.30±0.26
T3	2.25	3	10	86.67±0.68	0.985±0.005	948.00±10.58	3.43±0.01	12.33±0.32
T4	2	2	10	86.06±0.51	0.986±0.006	846.67±9.87	3.40±0.02	14.23±0.25
T5	2.25	3	7.5	85.19±0.59	0.985±0.005	1185.56±8.28	3.41±0.02	15.23±0.22
T6	2.25	3	5	86.05±0.58	0.986±0.004	1017.33±15.14	3.49±0.02	14.30±0.20
T7	2.5	3	7.5	86.64±1.04	0.981±0.005	956.67±13.61	3.50±0.01	14.40±0.20
T8	2	4	5	85.31±0.62	0.986±0.005	1014.33±9.29	3.51±0.04	15.13±0.21
T9	2.25	2	7.5	86.46±0.62	0.986±0.005	1044.67±15.28	3.50±0.02	14.43±0.31
T10	2	2	5	86.60±0.64	0.988±0.004	1156.33±10.60	3.39±0.01	13.63±0.40
T11	2.5	4	10	85.29±0.92	0.987±0.006	1218.00±11.14	3.40±0.02	15.37±0.25
T12	2.25	4	7.5	85.42±1.06	0.985±0.005	1069.01±13.45	3.37±0.01	15.20±0.36
T13	2.5	2	5	86.10±1.24	0.985±0.004	968.02±10.58	3.38±0.01	13.17±0.32
T14	2.5	4	5	85.65±1.06	0.983±0.005	942.01±11.14	3.37±0.02	14.90±0.26
T15	2.5	2	10	85.75±1.04	0.984±0.006	937.33±13.32	3.39±0.01	13.10±0.20
Significant effects (p-value) Biomass Inulin Time				0.287^ns^ 0.708^ns^ 0.246^ns^	0.195^ns^ 0.913^ns^ 0.913^ns^	0.002** 0.004** 0.000***	0.003** 0.06^ns^ 0.731^ns^	0.364^ns^ 0.000*** 0.000***

Results are reported as mean ± standard deviation. Different characters associated with process variables (biomass, inulin and time) indicate statistically significant differences (95%) at each response variable. ns: not significant. *p<0.05. **p<0.01.***p<0.001.

**Table 3:** Experimental design and analysis of variance (ANOVA) results for the analysis of cupuassu (*Theobroma grandiflorum*)-based cremogenate supplemented with *Lactobacillus casei* ATCC 393 and inulin. The table summarizes the experimental runs, formulation and processing factors, measured response variables, and the corresponding ANOVA outputs used to evaluate the significance of model terms and factor interactions.

Treatment	Biomass (%)	Inulin (%)	Time (min)	D10 (µm)	D50 (µm)	D90 (µm)	Zeta potential (mV)	CC (Log UFC/g)
T1	2	4	10	25.30±0.10	129.33±1.15	562.67±2.52	15.34±1.75	8.67±0.58
T2	2	3	7.5	19.50±0.10	114.00±1.00	502.67±1.15	16.71±0.97	8.33±1.53
T3	2.25	3	10	8.60±0.10	87.33±1.53	327.33±1.15	16.81±0.79	7.67±0.58
T4	2	2	10	7.70±0.10	89.00±1.00	336.67±1.53	14.57±1.33	8.33±1.15
T5	2.25	3	7.5	30.07±0.16	184.50±1.15	748.11±1.32	15.97±0.74	8.06±0.94
T6	2.25	3	5	35.77±0.15	226.00±1.00	850.33±1.53	16.18±1.12	8.33±0.58
T7	2.5	3	7.5	32.27±0.15	193.67±1.53	813.00±2.00	14.19±0.52	8.67±1.15
T8	2	4	5	36.50±0.10	221.00±1.00	922.67±1.53	15.66±1.02	11.33±0.58
T9	2.25	2	7.5	28.47±0.12	157.67±1.53	609.67±1.53	13.62±0.84	8.33±0.58
T10	2	2	5	34.30±0.10	196.33±1.53	715.33±1.53	15.01±0.68	9.33±1.53
T11	2.5	4	10	24.47±0.15	146.33±2.52	598.33±1.53	13.01±0.78	7.33±0.58
T12	2.25	4	7.5	25.27±0.15	138.00±1.00	547.33±2.08	16.97±1.37	8.33±0.58
T13	2.5	2	5	27.50±0.10	145.67±2.08	548.67±1.53	17.06±0.56	8.33±0.58
T14	2.5	4	5	26.60±0.10	142.33±1.53	566.67±1.53	17.62±0.81	7.33±1.15
T15	2.5	2	10	23.60±0.10	134.67±1.53	516.33±2.08	16.51±0.33	8.33±0.58
Significant effects (p-value) Biomass Inulin Time				0.512^ns^ 0.215^ns^ 0.000***	0.675^ns^ 0.209^ns^ 0.000***	0.986^ns^ 0.02* 0.000***	0.622^ns^ 0.372^ns^ 0.04*	0.005** 0.786^ns^ 0.078^ns^

Results are reported as mean ± standard deviation. Different characters associated with process variables (biomass, inulin and time) indicate statistically significant differences (95%) at each response variable. ns: not significant. *p<0.05. **p<0.01.***p<0.001.

The results obtained for the cupuassu-based cremogenate are summarized in Table 2, Table 3. Considering all evaluated responses, the physicochemical values were consistent with those previously reported for cupuassu juices and nectars [16], although they differed from cupuassu purees [18], which have been reported to contain substantially higher soluble solids contents, close to 60±1%. This difference may be mainly attributed to the use of added sugars and preservatives in puree formulations [18]. In the present dataset, soluble solids ranged from 12.33 to 15.37 °Brix and were significantly affected by inulin concentration and homogenization time (p<0.001; Table 2), whereas probiotic biomass did not show a significant effect (p>0.05). The increase in °Brix with higher inulin levels can be associated with the contribution of soluble solids from the prebiotic ingredient itself.

Some parameters showed relatively limited variation across the experimental design. Water activity ranged from 0.981 to 0.988, reflecting the high-moisture nature of the cupuassu-based matrix. However, according to Table 2, water activity was not significantly affected (p>0.05) by biomass, inulin concentration, or homogenization time. This indicates that, under the evaluated formulation conditions, the studied factors did not produce marked differences in the availability of water availability in the cremogenate. Moisture content ranged from 85.19 to 87.88% w.b. and was also not significantly affected (p>0.05) by the evaluated factors, confirming the relatively stable high-moisture character of the product.

Viscosity ranged from 846.67 to 1400.67 cP and was statistically significantly affected (p<0.05) by biomass, inulin concentration, and homogenization time. These results indicate that the rheological behavior of the cremogenate was sensitive to both formulation and processing conditions. In particular, the presence of inulin may contribute to changes in consistency due to its ability to modify texture and water-binding behavior in fruit-based systems. The pH values ranged from 3.37 to 3.53, indicating a stable acidic environment typical of fruit-based products [19]. Biomass had a significant effect on pH (p<0.05), whereas inulin concentration and homogenization time did not show significant effects (p>0.05). This acidic pH range may help limit the growth of spoilage microorganisms. However, its effect on probiotic stability should be interpreted only at the time of analysis, since shelf-life was not evaluated and should be addressed in future studies (See LIMITATION section).

Regarding the colloidal and particle-size properties, zeta-potential values ranged from 13.01 to 17.62 mV, indicating relatively low electrostatic stabilization compared with highly stable colloidal systems. Homogenization time was the only factor that significantly affected zeta potential, whereas biomass and inulin concentration were not significant (p>0.05). Particle-size distribution ranged from 7.70 to 36.50 µm for D10, 87.33 to 226.00 µm for D50, and 327.33 to 922.67 µm for D90. Homogenization time significantly affected D10, D50, and D90, confirming the expected effect of mechanical processing on particle-size reduction. Inulin concentration also significantly affected D90 (p<0.05), suggesting that the prebiotic ingredient may influence the distribution of larger particles in the cremogenate matrix.

Probiotic viability, expressed as log CFU/g, ranged from 7.33 to 11.33 log CFU/g. These values were above the minimum threshold commonly required for probiotic foods at the time of measurement. However, these results should not be interpreted as evidence of long-term probiotic stability, since refrigerated storage or shelf-life assays were not performed (See LIMITATION section). According to Table 3, probiotic viability was significantly affected (p<0.05) by biomass, whereas inulin concentration and homogenization time did not show significant effects (p>0.05). Therefore, the observed differences in colony counts can be interpreted as the response of *Lactobacillus casei* ATCC 393 under the specific formulation and processing conditions evaluated in this dataset.

**CremogenateAnalysis.m:** this MATLAB script was developed to perform response surface modeling, statistical screening, visualization, and numerical optimization of the experimental dataset obtained for the cupuassu-based cremogenate formulation. The dataset was imported from a **data.mat** file and organized according to the independent formulation and processing variables: homogenization time, probiotic biomass concentration, and inulin concentration. These factors were then associated with multiple dependent responses, including moisture content, water activity, viscosity, pH, °Brix, particle-size parameters (D10, D50 and D90), zeta potential, and CC. For each response variable, a second-order polynomial regression model (Eq. 1) was fitted using the “*fitlm”* function under a quadratic model structure. This approach enabled the estimation of linear, interaction, and quadratic effects of the independent variables, which is consistent with RSM. The resulting model coefficients, estimated parameters, p-values, root mean square error, and coefficient of determination were extracted to assess the explanatory capacity and statistical relevance of each fitted model (Table 4, Table 5).

(1) $$RV_{\text{pred}}=a_{0}+a_{1}T+a_{2}B+a_{3}I+a_{4}\left(T\;\times \;B\right)+a_{5}\left(T\;\times \;I\right)+a_{6}\left(B\;\times \;I\right)+a_{7}T^{2}+a_{8}B^{2}+a_{9}I^{2}$$

where T, B, and I represent the process variables time, biomass, and inulin concentration, respectively. The coefficients a_i_ correspond to the model parameters, where a_0_ represents the intercept term, a_1_ to a_3_ the linear effects, a_4_ to a_6_ the two-factor interaction effects, and a_7_ to a_9_ the quadratic effects of time, biomass, and inulin on the predicted response variable (RV_pred_). To evaluate the adequacy and robustness of the fitted regression models, a diagnostic assessment was performed using Studentized residuals, leverage values, and Cook’s distance [20]. The Studentized residuals were used to identify observations with unusually large deviations from the fitted model, considering a 95% confidence level. In this approach, observations may be flagged as potentially anomalous when the absolute Studentized residual exceeds the corresponding critical *t*-value (>|t^α/2^_Observations – ModelParameters − 2_|) [21]. Leverage values and Cook’s distance were also examined to identify observations with a potentially strong influence on the estimated regression coefficients. Nonetheless, in the present dataset, these diagnostic tools were used only for model validation.

**Table 4:** Statistical results of Multiple Linear Regression (MLR) models in the mathematical description of response variables as a function of time, biomass and inulin content.

Model terms	Response variables				
	Moisture content	Water activity	Viscosity (cP)	pH	°Brix
	Parameter (p-value)	Parameter (p-value)	Parameter (p-value)	Parameter (p-value)	Parameter (p-value)
b0	46.529***	1.084***	7137.357*	6.772***	–12.366^ns^
b1	0.012^ns^	–5.886 × 10^–3^***	8.525^ns^	–0.022^ns^	0.779^ns^
b2	24.776***	–0.041^ns^	–5104.186*	–3.947***	22.891*
b3	7.335***	–0.026***	–87.418^ns^	0.875***	–1.474^ns^
b4	–0.299***	2.586 × 10^–3^***	32.286^ns^	1.747 × 10^–4ns^	0.041^ns^
b5	–0.001^ns^	1.721 × 10^–4ns^	51.464***	1.756 × 10^–4ns^	1.667 × 10^–3ns^
b6	–2.527***	4.798 × 10^–3^***	–71.755^ns^	–0.115***	0.533^ns^
b7	0.051***	–2.832 × 10^–5ns^	–15.057***	1.421 × 10–3^ns^	–0.054*
b8	–3.164***	3.221 × 10^–3ns^	1060.441^ns^	0.923***	–5.631*
b9	–0.391***	2.495 × 10^–3^***	–10.684^ns^	–0.098***	0.173^ns^
F-values (p-value) Model Linear Non-linear	415.58*** 1065.52*** 90.63***	28.083*** 35.709*** 24.271***	10.502*** 13.215*** 9.142***	20.944*** 19.365*** 21.733***	19.567*** 48.555*** 5.072***
Goodness of fit					
RMSE (RU)	0.076	6.475 × 10^–3^	94.591	0.026	0.362
R^2^ (%)	99.342	91.321	69.234	83.221	80.118
R^2^_adj_	99.112	90.245	62.624	81.247	79.654
Lack of fit (p-value)	0.077^ns^	0.087^ns^	0.061^ns^	0.138^ns^	0.09^ns^
Pure error (sum squares)	0.117	8.055 × 10^–6^	1.495 × 10^5^	0.021	3.383

Different characters indicate statistically significant differences (95%) for each response variables as a function of model parameters. ns: not significant. *p<0.05. **p<0.01.***p<0.001.

**Table 5:** Statistical results of Multiple Linear Regression (MLR) models in the mathematical description of response variables as a function of time, biomass and inulin content.

Model terms	Response variables				
	D10 (µm)	D50 (µm)	D90 (µm)	Zeta potential (mV)	CC (log UFC/g)
	Parameter (p-value)	Parameter (p-value)	Parameter (p-value)	Parameter (p-value)	Parameter (p-value)
b0	–199.639^ns^	570.072^ns^	7611.906*	–78.132***	31.536^ns^
b1	–24.364***	–99.271***	–366.613***	–2.066*	–0.893^ns^
b2	238.366*	–144.812^ns^	–5600.059^ns^	66.875***	–20.535^ns^
b3	35.247***	108.301^ns^	486.01^ns^	15.351***	4.413*
b4	6.395***	34.238***	135.063***	–1.388***	0.397^ns^
b5	0.843***	1.535^ns^	4.158^ns^	–0.359***	–0.129*
b6	–10.606***	–31.182^ns^	–181.389*	–2.091***	–2.263***
b7	0.353^ns^	0.231^ns^	–0.093^ns^	0.411***	0.016^ns^
b8	–55.567*	–3.419^ns^	1140.278^ns^	–10.527*	4.795^ns^
b9	–2.657^ns^	–7.351^ns^	–10.083^ns^	–1.371***	0.266^ns^
F-values (p-value) Model Linear Non-linear	15.041*** 25.113*** 10.005***	7.539*** 16.541*** 5.039***	7.491*** 14.863*** 6.142***	26.586*** 10.957*** 34.401***	6.291*** 10.126*** 4.373***
Goodness of fit					
RMSE (RU)	3.727	27.214	110.159	0.542	0.724
R^2^ (%)	75.469	60.664	60.507	89.524	56.271
R^2^_adj_	74.954	60.142	59.985	88.743	55.056
Lack of fit (p-value)	0.41^ns^	0.68^ns^	0.61^ns^	0.686^ns^	0.062^ns^
Pure error (sum squares)	271.9	8502	98576	7.532	16.762

Different characters indicate statistically significant differences (95%) for each response variables as a function of model parameters. ns: not significant. *p<0.05. **p<0.01.***p<0.001.

The optimized response for each variable was obtained through constrained nonlinear optimization using the *fmincon* function in MATLAB 2023a (The MathWorks Inc., Natick, MA, USA) with the sequential quadratic programming algorithm. The objective function was defined from the fitted regression model, allowing the optimizer to search within the experimental factor bounds for the combination of homogenization time, biomass, and inulin concentration that maximized or minimized the predicted response, depending on the specific optimization criterion. Lower and upper bounds were defined directly from the experimental design space (Table 1), ensuring that the model-predicted optimum remained within the experimentally explored domain.

The optimization criterion was defined independently for each response variable. In all cases, the fitted regression models were used to identify the factor combination that maximized the predicted response within the experimental design space. This maximization-based approach was selected to explore the upper response limits predicted by the RSM models and to determine the formulation and processing conditions associated with the highest predicted value of each physicochemical, colloidal, particle-size, and microbiological response. It is important to note that these results do not represent a single global optimum formulation, since no weighted desirability function or simultaneous multi-response optimization was applied. Instead, the optimization results correspond to individual model-predicted maxima for each response variable. Therefore, the predicted conditions should be interpreted as response-specific computational optima rather than experimentally validated overall product-quality optima.

In addition, the provided models and MATLAB code allow users to modify the optimization criteria according to specific formulation or process requirements. Although all responses were maximized in the present study, the computational workflow can also be used to minimize, target, or constrain selected responses, depending on the desired product attributes. This flexibility enables users to explore how the predicted formulation conditions change when different optimization goals are applied, such as minimizing particle size or water activity, targeting a specific pH or viscosity range, or maximizing probiotic viability. Therefore, the code is not limited to the optimization scenario reported in this study, but provides a reusable tool for scenario analysis and decision support in future formulation studies.

The script also generated two-dimensional contour plots and three-dimensional response surface plots for each response variable. For visualization, homogenization time and biomass were represented as continuous grid variables, while inulin concentration was plotted at discrete experimental levels (Fig. 1). This graphical strategy allowed the interpretation of response behavior across the design space and facilitated the identification of regions predicted to provide improved physicochemical, colloidal, and microbiological performance. The model-predicted optimum was marked directly on the contour plots, together with the corresponding values of time, biomass, inulin, and the predicted response. Thus, the code provides a reproducible computational framework for data-driven optimization of the cupuassu cremogenate formulation. It integrates experimental design, quadratic response surface modeling, diagnostic assessment, statistical parameter extraction, graphical interpretation, and constrained numerical optimization. However, since no independent confirmation experiment was performed, the identified optimum should be interpreted as a model-predicted candidate formulation rather than an experimentally validated optimum. Future studies should include validation experiments to compare the predicted and experimentally obtained response values under the selected optimal conditions.

**Fig 1:**
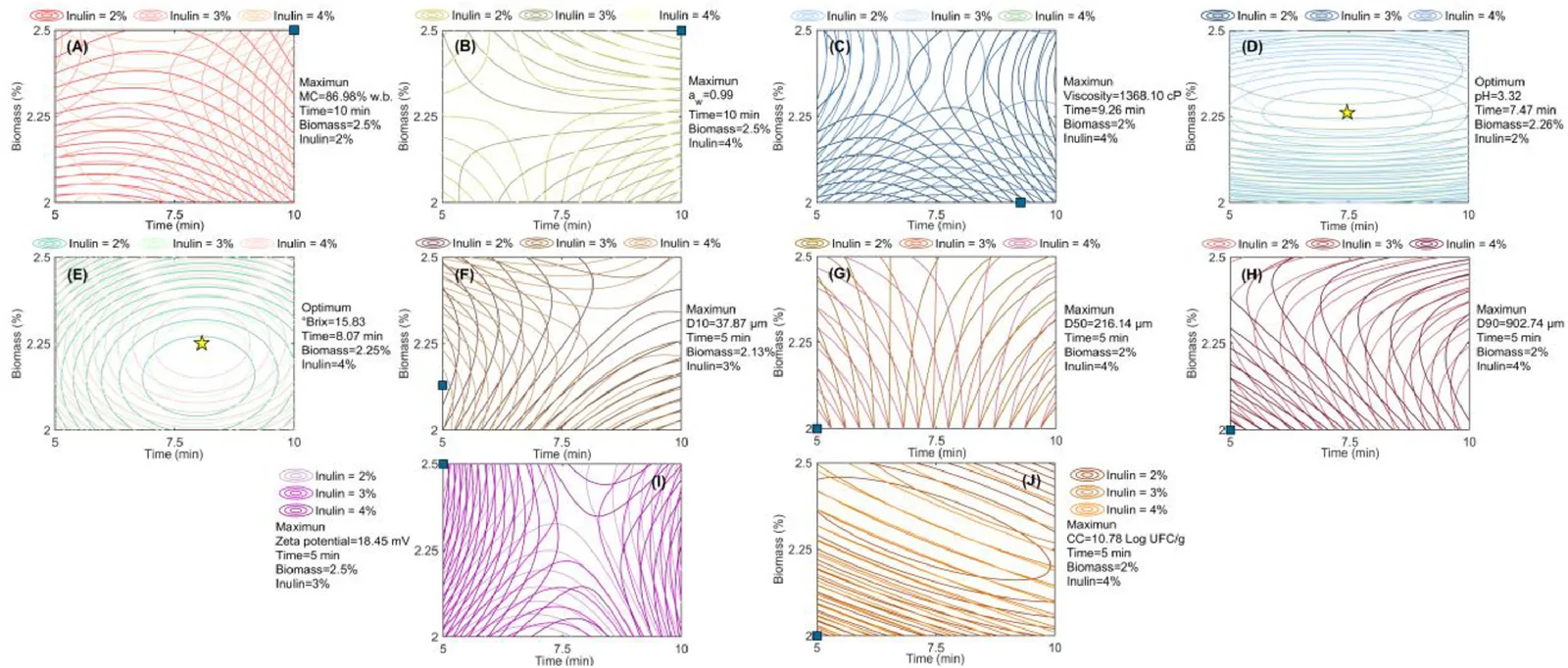
Surface responses showing the combined effects of probiotic biomass, homogenization time, and inulin concentration on the quality responses of cupuassu (*Theobroma grandiflorum*) cremogenates supplemented with *Lactobacillus casei* ATCC 393. The contour surfaces represent the influence of process variables on: moisture content (MC; A), water activity (a_w_; B), viscosity (C), pH (D), °Brix (E), D10 (F). D50 (G), D90 (H), zeta potential (I) and colony counting (CC; J).

### 3.1 Goodness-of-fit assessment of mathematical models

This step involves the assessment of predictive performance of adjusted models. This fact was achieved by the calculation of statistical metrics to assess how well the models (Eq. 1) fits the experimental response variables (RV_exp_). These metrics included the coefficient of determination (R^2^; Eq. 2), the adjusted coefficient of determination (R^2^_adj_; Eq. 3), the root mean square error (RMSE; Eq. 4). R^2^ indicates the proportion of variance in the experimental data explained by the model. It is calculated as the ratio of the residual sum of squares (RSS) to the total sum of squares (TSS). Furthermore, R^2^_adj_ indicates the proportion of variance explained by the model after accounting for the number of predictors included in the regression. Unlike R^2^, it penalizes the inclusion of unnecessary model terms, providing a more reliable measure of model performance, especially when comparing models with different numbers of variables. Higher R^2^ and R^2^_adj_ values indicate better predictive performance and a greater ability of the model to mathematically describe the response variables. Conversely, lower RMSE values indicate a better goodness of fit and lower prediction error during the modeling procedure [22].

(2) $$R^{2}\left(\%\right)=100-\frac{\text{RSS}}{\text{TSS}}=\;\frac{\sum_{i=1}^{N}{\left(RV_{\text{exp}}-\;RV_{\text{pred}}\right)}^{2}}{\sum_{i=1}^{N}{\left(RV_{\text{exp}}-\bar{RV_{\text{exp}}}\right)}^{2}}$$

(3) $$R_{\text{adj}}^{2}\left(\%\right)=100-\left(\frac{N-1}{N-M}\right)\left(100-R^{2}\right)$$

(4) $$\text{RMSE}\;\left(\text{RU}\right)=\sqrt{\frac{1}{N}\sum_{i=1}^{N}{\left(RV_{\text{exp}}-RV_{\text{pred}}\right)}^{2}}$$

Where RV_exp_ and RV_pred_ are the experimental and predicted response variables (moisture content, water activity, pH, °brix, particle size distribution D10, D50 and D90 zeta, potential and CC), N is the number of experimental data, M is the number of model parameters and RU are the units of each response variable.

The predictive performance of the fitted RSM models (Table 4, Table 5) varied among the evaluated response variables. While some responses were adequately described by the regression models (R^2^ and R^2^_adj_ >80), others, including CC, D50, D90, and viscosity, showed moderate explanatory ability. This indicates that the corresponding predicted maxima should not be interpreted as definitive or experimentally validated optimum conditions. Instead, these models should be used to describe response tendencies, explore the influence of formulation and processing variables, and identify regions of interest within the experimental design space. Accordingly, the optimization workflow is presented as an exploratory model-based tool for response surface interpretation and scenario analysis, rather than as a fully validated predictive optimization framework. The predicted maxima provide useful computational guidance for selecting candidate formulation conditions, but independent confirmation experiments are required to verify the agreement between predicted and experimentally obtained values.

## 4 Experimental Design, Materials and Methods

### 4.1 Raw materials

Fresh cupuassu fruits (*Theobroma grandiflorum* L.) were used as the primary raw material (Fig. 2A). Fruits were harvested at physiological maturity stage 2, characterized by natural peduncle detachment, according to the maturity classification described by [23]. The fruits were collected from the Macagual César Augusto Estrada Research Center, located at 250 m above sea level, approximately 22 km south of Florencia (Caquetá, Colombia), in cultivation zone 2 (1°29′55.3″ N, 75°39′45.7″ W). This region is representative of the Colombian Amazon piedmont and is recognized for the cultivation of native Amazonian fruit species. S20 inulin (Cosucra Groupe Warcoing SA, Brazil) was used as the prebiotic agent, while commercial stevia (Taeq®, Colombia) was incorporated as a non-caloric sweetener (Fig. 2A).

**Fig 2:**
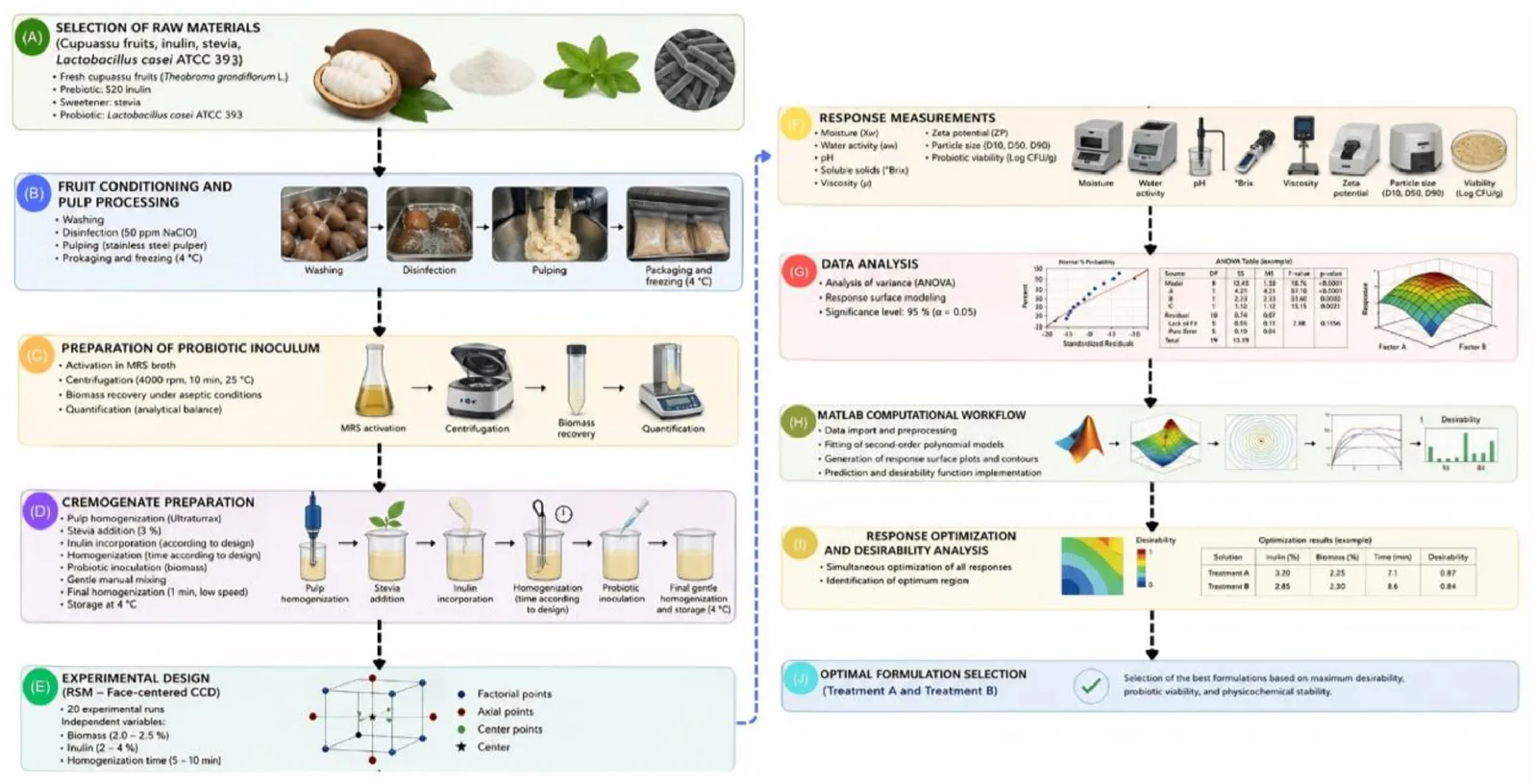
Schematic workflow of the experimental design and optimization strategy for the development of a functional cupuassu (*Theobroma grandiflorum*) cremogenate supplemented with *Lactobacillus casei* ATCC 393 and inulin. The workflow includes selection of raw materials, including cupuassu fruits, inulin, stevia, and probiotic culture (A); fruit conditioning and pulp processing (B); preparation of the probiotic inoculum (C); cremogenate formulation and homogenization (D); experimental design based on response surface methodology (RSM) using a face-centered central composite design (FCCD) (E); physicochemical, rheological, colloidal, and microbiological response measurements (F); statistical data analysis by analysis of variance (ANOVA) and response surface modeling (G); computational optimization and prediction (H); response optimization (I); and selection of the optimal formulation based on probiotic viability and overall product quality (J).

### 4.2 Probiotic culture preparation

A commercial freeze-dried probiotic culture corresponding to *Lactobacillus casei* ATCC 393 was used as the functional microbial strain. The inoculum was prepared by suspending the lyophilized culture in MRS broth (de Man, Rogosa and Sharpe; Conda, Spain). The culture was activated under microaerophilic conditions at 37°C for 4 h before biomass recovery. The suspension was centrifuged at 4000 rpm for 10 min at 25°C in sterile 50 mL Falcon tubes. Following centrifugation, the supernatant was discarded and the biomass was aseptically recovered under laminar flow conditions. The recovered cell pellet was weighed using an analytical balance (PB3002-S, Mettler Toledo, USA) and immediately incorporated into the formulation according to the biomass levels defined in the experimental design.

### 4.3 Cremogenate processing and formulation

#### 4.3.1 Pulp handling and hygienic preparation

Cupuassu fruits were washed and disinfected using a 50 ppm sodium hypochlorite solution. The pulp was then mechanically extracted using a stainless-steel pulping machine (300 kg/h, Ref. 01200515, JARINOX, No. 2 sieve). Pulp processing was performed at the pilot plant for fruit and vegetable processing of the Universidad de la Amazonia under controlled hygienic and biosafety conditions. The process comprised three operational stages: conditioning, separation, and preservation. The conditioning stage included raw material reception, selection, weighing, classification, cleaning, washing, disinfection, and rinsing. The separation stage included peeling, cutting, extraction, pulping, and weighing. The preservation stage consisted of packaging and freezing. All procedures were performed in compliance with standard biosafety protocols and appropriate sanitary handling practices. The microbiological and physicochemical quality of the raw pulp was previously established.

#### 4.3.2 Cremogenate preparation

The cupuassu pulp was initially homogenized using an Ultra-Turrax T18 homogenizer (IKA, 115 V) for 2 min at 15,500-15,600 rpm. Stevia (Taeq® Colombia) was then added at 3% (w/w), followed by incorporation of inulin (S20 inulin Cosucra, Groupe Warcoinng SA, Brasil) according to the experimental design. The mixture was subsequently homogenized for the time defined by each experimental treatment. To minimize mechanical damage to viable probiotic cells, the probiotic biomass was incorporated after the primary homogenization stage. The inoculum was first gently dispersed manually using a sterile instrument, followed by mild mechanical homogenization at 3500-3600 rpm for 1 min. The resulting cremogenate was immediately stored under refrigeration at 4°C until further analysis.

#### 4.3.3 Experimental design

The experimental design was established using a face-centered central composite design (as detailed in DATA DESCRIPTION section). A total of 15 treatment combinations were evaluated to assess the effects of three independent variables: probiotic biomass, inulin concentration, and homogenization time. The assessed levels were 2.0, 2.25, and 2.5% for probiotic biomass; 2, 3, and 4% for inulin and 5, 7.5, and 10 min for homogenization time. The center condition in the design was repeated six times to estimate pure error and support the evaluation of curvature in the fitted second-order polynomial models.

#### 4.3.4 Physicochemical, colloidal, and microbiological characterization

Moisture content was determined according to AOAC method 930.15, pH according to AOAC methods 942.15/970.21, and water activity according to AOAC 978.18. Soluble solids content, expressed as °Brix, was quantified according to AOAC 932.12 using a MA884 digital refractometer (Milwaukee Instruments, USA). All physicochemical measurements were performed at room conditions 26 ± 2°C and 50 ± 2%RH.

Viscosity was measured using a Brookfield rheometer equipped with spindle RV4. Before measurement, cremogenate samples were gently mixed to ensure homogeneity and allowed to equilibrate at 26 ± 2°C. Rotational speeds ranged from 0 to 100 rpm, and 20 measurements were recorded at 10 s intervals. The apparent viscosity was expressed in cP and calculated from the stable readings obtained during the measurement sequence.

Particle-size distribution was determined using a Mastersizer 3000 laser diffraction particle-size analyzer (Malvern Instruments Ltd., Worcestershire, UK). Before analysis, samples were homogenized and dispersed in distilled water until an adequate obscuration range was reached according to the instrument requirements. The characteristic particle diameters D10, D50, and D90 were recorded and expressed in µm. Each sample was measured in triplicate, and the results were reported as mean ± standard deviation. Zeta potential was measured using a Zetasizer Nano ZS90 (Malvern Instruments Ltd., UK) equipped with a capillary electrophoresis cell. Samples were diluted 1:50 in distilled water prior to analysis. The diluted samples were gently mixed to avoid foam formation and allowed to equilibrate before measurement. Measurements were performed at 26 ± 2°C, and zeta-potential values were obtained from electrophoretic mobility using the instrument software. Results were expressed as mean ± standard deviation.

Probiotic viability was quantified as colony-forming units per gram (CFU/g) by serial dilution and pour plating on MRS agar. For this analysis, 10 g of cremogenate was aseptically homogenized with 90 mL of sterile dilution medium, such as 0.1% peptone water or sterile saline solution, to obtain the initial dilution. Serial decimal dilutions (until 10^–6^) were then prepared using the same sterile diluent. An aliquot of 0.1 mL from appropriate dilutions was plated in MRS agar using the pour-plate method [9]. Plates were incubated under microaerophilic conditions at 37°C for 48 h. After incubation, plates containing 30-300 colonies were selected for the analysis [24]. Probiotic viability was calculated as CFU/g and expressed as log CFU/g. All analytical determinations were performed in triplicate (Fig. 2F).

### 4.4 Statistical analysis, modeling and optimization

Experimental data obtained from the response surface design (Table 2, Table 3) were statistically analyzed using Statgraphics Centurion XVIII (Manugistics, Inc., Rockville, MD, USA). The software was used to evaluate the effects of the independent variables (probiotic biomass, inulin concentration, and homogenization time) on the measured response variables, including physicochemical, rheological, colloidal, particle-size, and microbiological properties of the cupuassu-based cremogenate. Process optimization was carried out through RSM, which enabled the simultaneous evaluation of the linear, interaction, and quadratic effects of the independent variables on each response. Second-order polynomial models were fitted to the experimental data to describe the behavior of the system and to predict response values within the experimental domain. The adequacy and statistical significance of the fitted models were assessed by ANOVA, considering a confidence level of 95%. Model significance was determined based on the p-values of the regression terms, while the goodness of fit was evaluated using the R^2^ and RMSE. This statistical approach allowed identification of the most influential process variables and supported the prediction of optimal formulation conditions for maximizing probiotic viability while preserving desirable technological properties of the cremogenate (Fig. 2G to J).

## Limitations

This dataset represents an initial step toward the development of cupuassu-based probiotic cremogenates using inulin as a prebiotic ingredient. Further studies should be considered to expand its scope and applicability. In particular, other prebiotic polysaccharides, such as β-glucan, should be evaluated either individually or in combination with inulin to compare systems with different molecular structures and their effects on probiotic viability, viscosity, particle-size distribution, zeta potential, and colloidal stability. Moreover, validation using different fruit matrices, probiotic strains, formulation procedures, processing scales, and industrial conditions would be necessary to assess the broader applicability of the results. Finally, refrigerated storage assessment or shelf-life assays should be included to evaluate long-term probiotic survival, physicochemical stability, phase separation, and sensory changes during storage.

## Credit Author Statement

**Paola A. García-Rincón:** Conceptualization, Data curation, Formal analysis, Investigation, Methodology, Writing-original draft, Writing-review and editing. **Andrés F. Bahamon-Monje:** Conceptualization, Data curation, Formal analysis, Software, Investigation, Methodology, Writing-original draft, Writing-review and editing. **Gentil. A. Collazos-Escobar:** Data curation, Formal analysis, Investigation, Methodology, Software, Validation, Visualization, Writing-original draft, Writing-review and editing. **Nelson Gutiérrez-Guzmán:** Conceptualization, Project administration, Resources, Supervision, Validation, Writing-review and editing.

## Ethics Statement

The dataset acquired in this study did not involve human subjects, animal experiments, or data obtained from social media platforms.

## Data Availability

(Mendeley Data).Dataset and MATLAB-based computational tools for optimization of a probiotic Cupuassu (Theobroma grandiflorum) cremogenate (Original data)
